# A rapid review of opportunities and challenges in the implementation of social prescription interventions for addressing the unmet needs of individuals living with long-term chronic conditions

**DOI:** 10.1186/s12889-024-17736-2

**Published:** 2024-01-27

**Authors:** Uday Narayan Yadav, Grish Paudel, Saruna Ghimire, Bhushan Khatiwada, Ashmita Gurung, Shradha S. Parsekar, Sabuj Kanti Mistry

**Affiliations:** 1 National Centre for Aboriginal and Torres Strait Islander Well-Being Research, The Australian National University, Canberra, ACT Australia; 2 Centre for Primary Health Care and Equity, University of New South Wales, Sydney, Australia; 3 School of Health Medical and Applied Sciences, Central Queensland University, Sydney, Australia; 4 Department of Sociology & Gerontology and Scripps Gerontology Center, Miami University, Oxford, OH USA; 5 Department of Public Health, Torres University, Sydney, Australia; 6 Independent Freelance Consultant, Goa, India; 7 Public Health Evidence South Asia, Prasanna School of Public Health, Manipal, Karnataka, India; 8 School of Population Health, University of New South Wales, Sydney, Australia; 9 Department of Public Health, Daffodil International University, Dhaka, Bangladesh

**Keywords:** Long-term chronic conditions, Challenges, Opportunities, Social prescription, Primary health care

## Abstract

**Background:**

People with long-term chronic conditions often struggle to access and navigate complex health and social services. Social prescription (SP) interventions, a patient-centred approach, help individuals identify their holistic needs and increase access to non-clinical resources, thus leading to improved health and well-being. This review explores existing SP interventions for people with long-term chronic conditions and identifies the opportunities and challenges of implementing them in primary healthcare settings.

**Methods:**

This rapid review followed the Preferred Reporting Items for Systematic Review and Meta-analysis guidelines and searched relevant articles in three databases (PubMed/MEDLINE, EMBASE, and Web of Science) by using subject headings and keywords combined with Boolean operators. The search encompassed articles published between January 2010 and June 2023. Two authors independently conducted study screening and data abstraction using predefined criteria. A descriptive synthesis process using content analysis was performed to summarise the literature.

**Results:**

Fifteen studies were included, with all but one conducted in the United Kingdom, and revealed that social prescribers help guide patients with long-term chronic conditions to various local initiatives related to health and social needs. Effective implementation of SP interventions relies on building strong relationships between social prescribers and patients, characterised by trust, empathy, and effective communication. A holistic approach to addressing the unmet needs of people with long-term chronic conditions, digital technology utilisation, competent social prescribers, collaborative healthcare partnerships, clinical leadership, and access to local resources are all vital components of successful SP intervention. However, the implementation of SP interventions faces numerous challenges, including accessibility and utilisation barriers, communication gaps, staffing issues, an unsupportive work environment, inadequate training, lack of awareness, time management struggles, coordination and collaboration difficulties, and resource constraints.

**Conclusion:**

The present review emphasises the importance of addressing the holistic needs of people with long-term chronic conditions through collaboration and coordination, training of social prescribers, community connections, availability of local resources, and primary care leadership to ensure successful interventions, ultimately leading to improved patient health and well-being outcomes. This study calls for the need to develop or utilise appropriate tools that can capture people's holistic needs, as well as an implementation framework to guide future contextual SP interventions.

**Supplementary Information:**

The online version contains supplementary material available at 10.1186/s12889-024-17736-2.

## Background

Long-term chronic conditions, such as diabetes and cardiovascular diseases, are the leading cause of preventable morbidity and mortality worldwide [1]. People with long-term chronic conditions experience a significant disparity linked to social and cultural determinants, including unemployment, low education levels and income, poor support for addressing their holistic needs, as well as challenges in accessing and navigating diverse health and social services. All of these determinants are known to contribute to the development and progression of adverse health outcomes. Health promotion and prevention of disease progression is a crucial component of long-term chronic condition management, and social prescribing (SP) can play a key role in both preventing progression and addressing the complex and intertwined health and social inequalities faced by individuals with long-term conditions. In order to reduce disparities, public health programs and social services need to be integrated to address the holistic needs of people with long-term chronic conditions. The World Health Organization (WHO) [2] has defined social prescribing as “a means of connecting patients to a range of non-medical services in the community to improve their health and well-being.” Social prescribing (SP) is gaining global popularity, as evidenced by the WHO’s interest. However, it’s worth noting that the majority of these interventions have been limited to high-income countries [3]. Social prescribing, also known as community referral, is a community-based, person-centred holistic approach to help and support individuals to identify their healthcare needs and to take actions to improve health and well-being, thereby reducing the demand for secondary health care services [4–6]. It has the potential to positively impact the health and well-being of individuals dealing with long-term chronic conditions, social isolation, and complex care needs [4, 6].

Several social prescribing models, such as art therapy, green prescription and physical activity programs, are delivered through different platforms to accommodate the diverse needs of communities and care settings [2]. An emerging model for delivering SP programs is through primary health care (PHC) settings, where PHC providers such as general practitioners (GPs) or practice nurses refer patients to a specialised link worker (also known as a community connector, navigator, or health adviser). These link workers identify individual’s holistic needs, match them with fitting services, co-design personalised self-management plans, and encourage healthy behaviours to promote their well-being [2, 4, 6]. Social prescribing interventions have been applied to diverse groups, including those with mental health conditions [6, 7]. Particularly within the realm of long-term chronic conditions, SP programs have been extended to a wide range of conditions, such as diabetes, cardiovascular diseases, respiratory diseases, and multimorbidity, all facilitated through PHC settings [8–10].

Living with long-term chronic conditions can limit an individual's opportunities to participate in social engagement activities, practice healthy lifestyle behaviours, and develop a financial crisis management plan [6]. Consequently, these limitations can significantly impact the health and well-being of the individual. In line with this, the WHO suggests that social prescribing is an important approach for addressing the holistic needs of people with long-term chronic conditions by integrating both medical and non-medical services required to maintain a healthy life [11].

Although SP models are increasingly used to prevent and manage long-term chronic conditions in PHC settings [10, 12, 13], there is a lack of a comprehensive review that explores existing SP programs for long-term chronic conditions, including the potential facilitators and barriers. Therefore, this review aimed to address this knowledge gap by exploring the existing SP programs for people with long-term chronic conditions and identify the opportunities and challenges of implementing such initiatives in PHC settings.

### Methods

This rapid review was conducted in accordance with the Preferred Reporting Items for Systematic Reviews and Meta-Analyses extension for Scoping Reviews guideline [14]. We searched three databases (MEDLINE via PubMed, EMBASE and Web of Science) to identify relevant English language studies published between January 2010 and June 2023.

### Search strategy

The search methodology applied in each database is provided as a Supplementary file 1. Briefly, the literature search was performed using a combination of subject headings and keywords pertinent to “social prescription,” “chronic disease,” and “primary health care” intertwined through the Boolean operators “OR” and “AND.” Furthermore, within each database, additional criteria for inclusion and exclusion were applied to restrict the studies exclusively to original studies (i.e., primary interventional studies, observational studies, implementation research, mixed methods studies, and qualitative evaluation). Letters to the editor, commentaries, editorials, and reviews were excluded. Research focusing on long-term chronic conditions such as cardiovascular diseases, diabetes, chronic obstructive pulmonary diseases (COPD), bronchial asthma, arthritis, hypertension, and obesity were included. Conversely, studies where social referral interventions solely targeted people with mental health conditions, terminal illnesses like cancer, and infectious diseases such as tuberculosis and HIV infection were deemed ineligible for inclusion.

Furthermore, based on the referral mechanism, we included studies in which individuals with long-term chronic conditions were linked to a link worker via PHC. These link workers then directed them to a range of clinical and non-clinical services. The spectrum of link workers encompassed roles spanning social workers, health promoters, Indigenous health workers, community health workers, health educators, or patient navigators, all situated within the PHC settings. Throughout this review, we utilise the term "social prescribers" to encompass the various titles for these link workers across the studies. Studies in which social prescribers operated beyond PHC settings or when interventions were not specifically tailored to individuals with long-term chronic conditions were excluded. Any initiatives being delivered as part of the SP program that provided information pertaining to intervention delivery, challenges and facilitators with regard to the abovementioned long-term chronic conditions were included. SP intervention could be delivered face-to-face or over the telephone.

### Screening and study selection

The search results from each database were imported to Endnote software (version 20) [15] to facilitate subsequent transfer to the Covidence platform. Within Covidence, duplicate entries were eliminated, and each record underwent initial screening based on its title and abstract, followed by a comprehensive full-text assessment [16]. Two reviewers (UNY and a research officer SB) independently assessed the titles and abstracts of potential studies to determine their eligibility based on the inclusion and exclusion criteria. Studies identified as potentially meeting the eligibility criteria during the title and abstract screening phase were then subjected to full-text screening by the same two reviewers (UNY and a research officer SB). Any discrepancies that emerged during the screening process were resolved through discussions among the team members.

### Data extraction and synthesis

The relevant data from the included studies were extracted by a research officer SB, UNY and GP in Microsoft Excel spreadsheets and encompassed various aspects of the studies, including their title, authors, publication year, study settings, participant characteristics, study objectives, study design and details regarding social prescription, along with barriers and facilitators encountered during the implementation of SP (Supplementary file 2). To ensure precision, the extracted data were cross-verified by one other research team member. The extracted data were analysed following a descriptive synthesis process using content analysis [17].

## Results

### Study selection

**Fig. 1:**
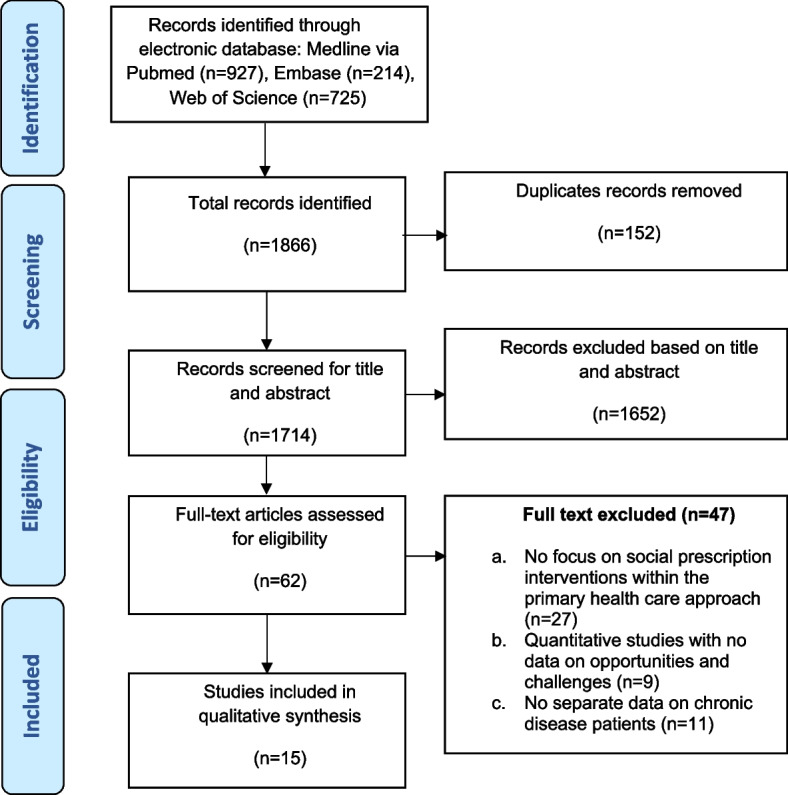
PRISMA flow diagram (2009) for reporting systematic review and meta-analysis

The initial search produced a total of 1866 records. In the subsequent phase, 152 duplicates were eliminated, leaving 1714 records for title and abstract screening. Out of these, 62 articles met the eligibility criteria based on abstract and title screening and advanced to the full-text screening stage. Among these, 47 articles were excluded because their focus was not on PHC settings or individuals with pertinent long-term chronic conditions, and/or they lacked relevant data on social prescription. This led to a final inclusion of 15 articles for this review. The details of the screening and selection process are illustrated in Fig. 1.

### Study characteristics and setting

Among the fifteen studies that were included, five were published between 2017 and 2019, six in 2021, two in 2022, and one in 2023. With the exception of one study conducted in Australia [18], all the included studies took place in the United Kingdom (UK). The encompassed studies exhibited a range of research designs, comprising a cohort study [19], cross-sectional study [20], pragmatic study [21], mixed-method studies [8, 18, 22, 23] and qualitative studies [12, 24–30]. Table 1 presents the characteristics of these studies.

**Table 1:** Characteristics of the included studies

Study	Study objective	Study design	Study setting	Study population/participants	Social referral medium
Carnes et al., 2017 [7]	i) To assess the effect of service on mental well-being and primary health care resource use and ii) To assess whether the service could be implemented as intended	Mixed methods approach	22 primary care general practices in London, UK	487 patients in GPs who were frequent attenders and, or socially isolated. People were not referred if they were in acute crisis, at risk to self and/or others, had uncontrolled addictions or mental health problems	GP referred the patients to a social prescribing coordinator
Moffatt et al., 2017 [11]	To describe the experiences of patients with long-term conditions involved in the link worker social prescribing programme	Qualitative study	Inner city areas in West Newcastle, UK (Most socioeconomically deprived areas)	30 patients aged 40–74 years with one or more long term conditions	GP, practice nurse, and healthcare assistants referred to a trained link worker
Mistry et al., 2023 [16]	To explore the feasibility and acceptability of involving Bilingual Community Navigators (BCNs) in the general practice setting in Australia	Mixed-method design	Sydney, Australia	95 patients attending the general practices; 3 practice staffs; and 3 trained community health workers (BCNs)	GP referred patients to the navigator
Wildman & Wildman 2021 [17]	To determine whether a UK’s NHS Community Health Worker social prescribing program was associated with improved HbA1c levels among type 2 diabetes patients	Cohort study	High socioeconomic deprivation areas in Northeast England	8086 adults aged 40 to 74 years with type 2 diabetes	Primary care practitioner referred the patient to a link worker(CHW)
Tierney, Wong & Mahtani 2019 [18]	To explore how 'care navigation' is interpreted and currently implemented by clinical commissioning groups (CCGs) in England	Cross-sectional survey	All clinical commissioning groups England	147 CCGs who provided info on care navigation	Social referral medium varied and mostly included self-transfers by patients or referrals by healthcare professionals (GPs), and trained receptionists. In some CCGs, multidisciplinary teams like accident & emergencies workers, hospitals, voluntary and community sectors, and others like police, fire brigades, ambulance services, libraries, pop-up clinics in supermarkets, rehabilitation teams, dementia teams, mental health teams, carers and family members made the referrals
Kiely et al. 2021 [19]	To test the effectiveness of primary care-based link workers providing social prescribing in improving health outcomes for people with multimorbidity	Pragmatic study	General practices in deprived areas in Ireland	12 patients with multimorbidity who attend general practices in deprived areas in Ireland. 6 patients, 1 link worker and 2 GPs included in the evaluation of trial’s acceptability and feasibility	Patients were referred to the link worker by the GPs
Gibson et al., 2021 [20]	To explore the complex social contexts in which social prescribing is delivered	Qualitative	Ethnically and socially mixed urban area of North of England	Four clients aged between 40 and 74 years with at least one of eight qualifying long-term health conditions from primary care	Patients were referred by their GPs to Ways to Wellness program
Frostick & Bertotti 2021 [21]	To identify the training, skills and experience of social prescribing Link Workers, working with patients with long-term conditions	Qualitative study	Three social prescribing schemes based within London and the southeast of England	13 experienced link workers actively working on the social prescribing scheme and had been there for six months or more	Referrals were made by GPs
Hanlon et al., 2021 [22]	To explore the utility of self-determination theory in improving well-being by connecting patients to appropriate community resources	Qualitative study	General practices based on socioeconomically deprived areas of Glasgow, Scotland	12 patients with physical, psychological, or social problems that the GPs or practice nurse felt might benefit from seeing the Community Link Practitioners (CLPs)	General practitioners or practice nurse referred patients to CLPs. Also, some patients self-referred to CLPs
Morris et al., 2022 [23]	To explore how people with LTCs managed their health and well-being under social distancing restrictions and self-isolation during the first wave of the COVID-19 pandemic	Qualitative study	Northeast England (ethnically diverse urban locality including urban fringes)	44 people with one or more long-term conditions who were already part of a social prescribing intervention evaluation (Wildman et al., 2019, Moffatt et al., 2019 and Gibson et al., 2021)	Patients were referred by their primary care practitioner to Ways to Wellness program and assigned to a trained link worker
Morris et al., 2022 [24]	To describe changes to the social prescribing service during the first wave of the COVID-19 pandemic	Qualitative study	Ethnically diverse urban locality (including urban fringes) in Northeast England	44 community-dwelling adults aged 40–74 with at least one of the following long-term conditions: diabetes type 1 and 2, heart failure, coronary heart disease, epilepsy, osteoporosis, asthma, and/or chronic obstructive pulmonary disease with or without anxiety and/or depression. Additionally, 5 link workers and 8 service managerial staff	Patients were referred by their GPs to Ways to Wellness program and assigned to a link worker
Wildman et al., 2019 [25]	To explore experiences of social prescribing among people with long term conditions	Qualitative study	Socioeconomically deprived area of Northeast England	24 individual aged 40 to 74 years with long-term conditions who were users of the link worker social prescribing service	The primary care practitioner referred the patient to a link worker
Chng et al., 2021 [26]	To explore the implementation process of social prescription approach involving primary care-based 'link workers'	Qualitative study	Seven general practices in deprived areas of Glasgow, Scotland over two years period	Participants were practice staff with responsibility for leading the Link Worker Programme (lead General Practitioners, Community Link Practitioners, and practice managers) and community organisation workers identified by Community Link Practitioners. Number of participants varied in different phases	Referrals were made by General Practitioners
Hazeldine et.al., 2021 [27]	To describe the experiences of early implementation of link worker social prescribing; to assess how this series of relationships functions; and identify the key barriers and facilitators experienced on the ground	Qualitative study	Southwest of England	11 link workers, 2 link worker managers and 1 counsellor	GPs referred patients to link workers
Wildman et al., 2019 [28]	To explore link workers self-definitions of their roles in social prescribing and self-identified skills and qualities necessary for effective client linkage	Qualitative study	Social prescribing scheme in a socioeconomically deprived area of Northeast England	15 participants, aged 40 to 74 years, with long-term conditions who were SP service users participated in FGD	Patients were referred to the CLP by GPs, practice staff or could self-refer

The included studies showcased a wide array of study settings. Most of the studies were conducted within areas marked by socio-economic deprivation [8, 12, 21, 24, 27, 28]. One study was conducted on PHC practices within the National Health Services (NHS) [19], while another study was housed within NHS’s Clinical Commissioning groups in England [20]. The Australian study [18] specifically targeted individuals from culturally and linguistically diverse backgrounds, with a particular emphasis on the Chinese and Samoan communities residing in Sydney. Additionally, three of the studies [22, 25, 26] were executed in ethnically diverse and urban localities, including urban fringes. One study [29] was conducted within marginalised communities, while three other studies [18, 23, 30] occurred in communities displaying diversity in ethnicity, gender, and age.

### Operational definition

The term social prescriber used in this study represents diverse titles held within the included studies, such as community link practitioners [24, 28], link workers [8, 12, 19, 21–27], support workers [20], social prescribing coordinator [8] and bilingual community navigators [18].

### Social prescription: intended recipients, social prescribers, referral and follow-up pathways

The intended recipients of social referrals displayed variability across the studies, with specific criteria employed to identify individuals who could potentially benefit from the programs. Most of the studies explicitly focused on individuals aged between 40 and 74 years who had long-term chronic conditions [12, 18, 20, 25–27]. The long-term chronic conditions reported encompassed type 2 diabetes, COPD, cardiovascular diseases, chronic liver conditions, respiratory diseases, and multimorbidity.

In all of the studies, the pathways for referral to social prescribers were made by GPs within the PHC setting [8, 12, 18–30]. In two studies [24, 30], individuals either self-referred or were referred by general practice staff. One study [20] under the NHS Clinical Commissioning Groups program employed a diverse range of approaches for social prescription, including self-referral and referrals through trained receptionists, accident and emergency workers, hospitals, volunteers, community members etc.

In terms of follow-up, in four studies [12, 18, 22, 26], social prescribers established connections with individuals by scheduling appointments at GP practices, cafes, community centres, participants' homes, council centres, and sometimes via telephone, email, and text. In one study [25], individuals involved in the SP intervention maintained contact with social prescribers through digital platforms and telephones.

### Existing SP programs for people living with long-term chronic conditions

Social prescribers played a pivotal role in supporting and directing individuals with long-term chronic conditions towards a variety of programs designed to enhance interactions, motivation for action plans, and access to essential services [8, 18, 22, 24]. These efforts assisted individuals in rediscovering past interests and fostering the formation of groups centred around new shared passions [22, 24]. One illustrative example of such initiatives was connecting individuals with diabetes to local gym facilities, weight management programs, and diverse activity groups like walking, healthy eating, and breathing exercises, effectively promoting a healthy lifestyle [12, 21, 22, 27]. Furthermore, individuals with long-term chronic conditions dealing with mental health challenges and social isolation engaged in an array of community-based activities such as gardening, fishing, crafts, and participation in voluntary groups, alongside arts-focused endeavours like choirs and art therapy [12, 22, 27].

**Fig. 2:**
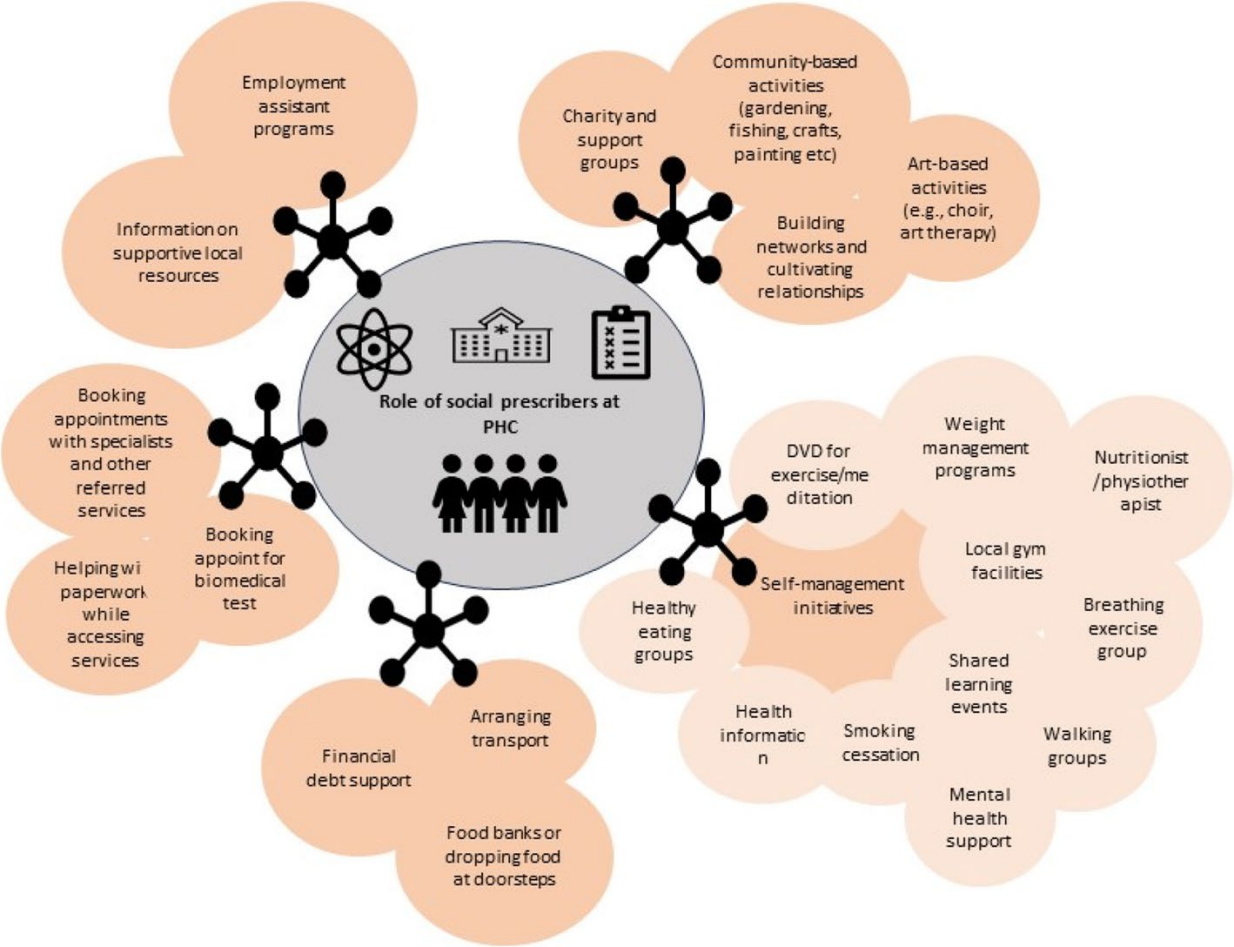
Mapping of social prescription intervention

Social prescribers aided with medical appointments, paperwork, and offered information about local resources, including social benefits and transportation [18]. They provided emotional support through active listening and empathetic understanding [18]. Diverse strategies were employed to help those facing financial challenges, including providing financial guidance, connecting individuals with charitable and support groups, offering welfare advice, and providing employment assistance [12, 22, 27]. Additionally, social prescribers linked economically disadvantaged individuals with food banks, supplying food vouchers [22]. Amid the COVID-19 pandemic, social prescribers leveraged digital tools, including telephone calls and social media, to distribute exercise resources and coordinate food delivery [25, 26]. In addition to patient-centred work, social prescribers developed referral pathways by actively building networks and collaborating with local organisations and organised shared learning events to strengthen both new and existing community connections [8, 18, 28]. Figure 2 illustrates the SP interventions catering to people living with long-term chronic conditions.

### Opportunities and challenges for implementing social prescribing intervention

**Fig. 3:**
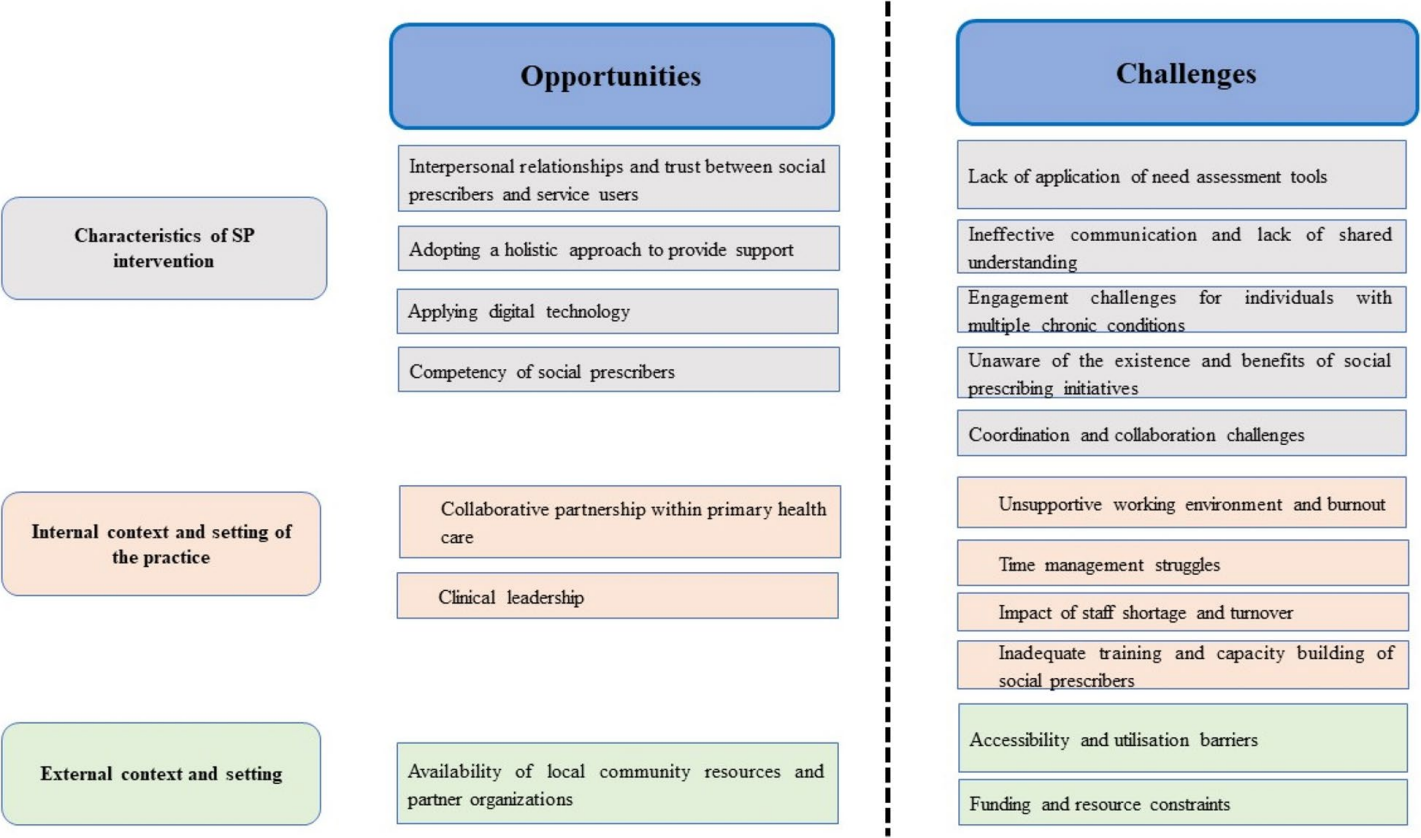
Opportunities and challenges for implementing social prescription intervention for people with chronic disease

The opportunities and the challenges of enacting SP programs related to the implementation of SP interventions in the included studies were categorised into three overarching themes applying domains of Consolidated Framework for Implementation Research [31]: Characteristics of SP intervention, Internal context and setting of the practice and External context and setting. The Fig. 3 depicts all three themes under which opportunities and challengues are presented along with subsequent sections that delve into comprehensive explanations of each sub-theme.

Within the framework of this review, opportunities refer to the set of circumstances or factors that enabled the implementation and utilisation of SP programs. Similarly, challenges are denoted as the issues or obstacles encountered by stakeholders (including healthcare providers and patients) while implementing and using SP programs.

## Characteristics of SP intervention

### Opportunities

#### Interpersonal relationships and trust between social prescribers and service users

Establishing a strong and trusted relationship between social prescribers and individuals with long-term chronic conditions was pivotal for effective social prescribing and greater engagement in SP programs [18, 21, 23]. Social prescribers served as key advocates, addressed the clinical and non-clinical needs of those with long-term chronic conditions [12, 18, 20, 22, 24–27], motivated and boosted confidence, and promoted referrals to necessary health and well-being services [8, 12, 18, 19, 22, 24–27]. These prescribers adeptly cultivated open and trustworthy bonds with service users, even offering counselling during bereavement [18, 24, 26] and promoting social engagement [22]. Notably, their strong interpersonal skills and attentive listening [18, 27] fostered trust with service users, resulting in increased satisfaction and reduced complaints. Key attributes such as face-to-face interactions, interaction quality, trustworthiness, friendliness, empathy, non-judgmental demeanour, motivational support, and clear communication of potential risks and benefits by social prescribers were valuable facilitators of SP programs [8, 19, 24]. Furthermore, the prompt adaptation of interventions to assist vulnerable populations during the COVID-19 pandemic not only bolstered social prescribers' relationships with patients but also with health care professionals and community organisations [23, 26].

Addressing the feedback received from individuals with long-term chronic conditions emerged as a pivotal factor in constructing trust and nurturing a value-centred relationship between social prescribers and service users [23]. Community-based connections and local familiarity played a crucial role in boosting social capital and encouraging proactive engagement in activities, thereby motivating commitment toward investing in health [22]. A study from Australia [18] highlighted the importance of recruiting and involving social prescribers from the local community to cultivate patient trust.

#### Adopting a holistic approach to provide support

Participants noted that social prescribers tailored services to their individual needs [12, 19, 22, 24–27], resulting in feelings of satisfaction, enjoyment, and motivation from engaging in SP interventions [18, 19, 24, 27]. Moreover, offering motivation, encouragement, and support to access specialised health services fortified individuals' confidence and ability to manage their health issues [18, 27]. For those with diabetes, prescribers fostered confidence through consistent engagement in gym and walking groups, blood sugar management via food choices and healthy eating advice, leading to physical health improvement [12, 22, 24]. Furthermore, a systematic approach involving needs assessment, motivational interviewing, connection to local preventive health services (e.g., local physical activity groups and specialised dieticians) and action planning proved effective in enhancing healthy lifestyles and self-care among patients [12, 18, 21, 26].

During COVID-19 pandemic, the provision of food bank vouchers from social prescribers increased the access to government financial support, aiding the maintenance of a healthy lifestyle [25]. Additionally, social and emotional support, bereavement counselling, addressing traumatic disruptions, and health condition follow-ups by social prescribers played a crucial role in prioritising health during the pandemic [18, 26]. Similarly, social prescribers offered referrals to diverse community initiatives such as welfare rights, employment support, housing advice, and health and lifestyle support, potentially elevating people's living standards [18, 19, 27]. In Australia, social prescribers guided individuals with long-term chronic conditions in understanding the Australian health system, booking appointments, preparing for attendance, and ensuring necessary follow-ups [18]. A study in the England underscored how favourable economic positions of individuals with long-term chronic conditions facilitated engagement in interventions, allowing more time for managing long-term chronic conditions [22].

#### Application of digital technology

Using digital platforms and social media, such as telephone, email, and text services, social prescribers facilitated SP programs [12, 25]. For example, people followed DVD instructions via digital and social media for physical activity [25]. Amid the COVID-19 pandemic, service users connected with social prescribers via phone, email, or social media, often having video chats to discuss their needs. This depicts the important role of social prescribers in offering emotional support via digital platforms, especially for those grappling with complex health issues during challenging times [18, 24, 26].

#### Competency of social prescribers

Social prescribers underwent training aimed at establishing connections and networks, with variations in training type and duration across studies. In one study, social prescribers received training in behaviour change methods to deliver personalised services based on patient goals and priorities, using a holistic well-being approach [12]. Other approaches included individual assessment, motivational interviews, action plans, facilitating access to community services, fostering trust-based bonds, promoting behavioural change for a healthy lifestyle, and imparting techniques for decision-making [8, 12, 18, 21, 23, 27, 30]. Training also encompassed mental health first aid and motivational interviewing to support long-term chronic disease condition individuals with psychological issues [23] as well as arranging specialised appointments [18, 20]. Additionally, support for social prescribers included monthly manager supervision, interactive learning sessions, logbook maintenance, informal knowledge sharing, and peer discussions for knowledge updates and upkeep of the community directory of activities and resources [18, 29]. Social prescribers' previous work experience with the community facilitated program implementation in some instances [18, 29]. Seven studies mentioned about the use of social needs screening proprietary tool “Well-being Star” on various domains such as lifestyle, self-care, symptom management, work and volunteering, money, living conditions, social relationships, and mental well-being by social prescribers [12, 19, 22, 25–27, 30].

### Challenges

#### Lack of application of need assessment tools

In general, a lack of clarity regarding screening tools for evaluating social needs and guiding appropriate social referrals was observed across the majority of studies. A solitary study from Australia [18] made a reference to the utilisation of needs assessment/problem identification by social prescribers; however, the specific types of needs assessed in this context remained unclear. The struggle of social prescribers to effectively refer service users to activities and resources stemmed from limited information about local options [18]. Multiple studies also emphasised the need for a clear implementation plan and process indicators, facilitating the effective delivery and evaluation of SP programs [18, 20, 28, 29].

#### Ineffective communication and lack of shared understanding

Communication gaps, including irregular or absent contact with social prescribers [25–27], a lack of understanding of SP services among health care providers, social prescribers, and patients [8, 18, 23], and insufficient communication from the local government regarding interventions [25] hampered the utilisation of SP services. Participants also emphasised the significance of in-person interactions as more enjoyable than remote communication via phones or text, the latter leading to decreased motivation for engagement in the interventions [26]. Social prescribers noted that referred patients often lacked awareness of the reasons for seeing a social prescriber [18] and faced challenges in building rapport and practicing therapeutic skills remotely [26]. In three studies [18, 23, 29], social prescribers expressed dissatisfaction with unclear information received from GPs, potentially leading to inappropriate referrals and decreased effectiveness of the intervention. One study [18] highlighted the importance of GPs conducting more thorough patient screenings in relation to required services before making referrals to social prescribers to prevent overwhelming workloads and increased waiting times for services.

#### Engagement challenges for individuals with multiple long-term chronic conditions

Among individuals dealing with multiple long-term chronic conditions and compromised mental well-being, reasons for disengagement were frequently observed [12, 19, 27]. This group faced a range of challenges, including communication-related anxiety [19, 24], lack of confidence [19, 27], social isolation [12, 24, 27, 29], and a perceived sense of dependence on social prescribers. For those with multiple chronic conditions, maintaining consistent participation in activities posed a considerable challenge, resulting in increased absenteeism and higher dropout rates [12].

#### Unaware of the existence and benefits of SP programs

Individuals with long-term chronic conditions felt overwhelmed by referrals, citing concerns about their appropriateness and time commitments [8, 26]. In two separate studies [8, 26], certain participants with long-term chronic conditions were unaware of the existence and benefits of SP programs. In another study, many of the referred individuals did not receive the necessary support due to conflicting priorities and the considerable distances to service centres. In one study [21], patients misunderstood the term "prescribing" as medication-related, causing confusion.

#### Coordination and collaboration challenges

Studies show that insufficient collaboration and coordination among service providers, link workers, and clients have hindered the successful implementation of SP programs [18, 19, 27]. Chng et al. emphasised problematic team dynamics, especially between administrative and GP staff, along with difficulties in maintaining connections with community organisations, impacting the effectiveness of social prescribing programs [28]. GPs also noted a lack of coordination between administration and health professionals, including social prescribers, impeding the development of a contextually suitable strategic framework [28].

## Internal context and setting of the practice

### Opportunities

#### Collaborative partnership with primary health care

A shared understanding of the roles of social prescribers among GPs and service staff helped social prescribers to effectively connect those with chronic conditions to non-clinical services [18, 28]. Pre-existing supportive informal networks and healthy team relationships among practice staff motivated social prescribers, fostering efficient collaborative work [18, 28]. Many practices often allocated dedicated spaces (practice rooms, waiting rooms for patients, and logistical arrangements) for social prescribers within their practice and invited prescribers to staff meetings, further enhancing effective collaborative work [18, 21–23, 29]. In many studies, GPs' high commitment to identifying and connecting needy patients with social prescribers were crucial for the success of SP programs [18, 21]. This commitment significantly shaped the team culture within practices, integrating social prescribers effectively into the practice team. Notably, one study highlighted that providing social prescribers access to client management databases enabled them to gather patient-related information and prepare to offer support [21].

#### Clinical leadership

Social prescribers and health care providers highlighted that supportive leadership from general practice managers and commissioners had an impact on the delivery of social prescribing. These leaders contributed to aspects like recruiting social prescribers and establishing referral pathways for SP programs [12, 24, 27, 29].

### Challenges

#### Unsupportive working environment and burnout

Social prescribers noted an unsupportive working environment, insufficient support from practice staff, limited unity in the leadership, strained team relationships, and fewer learning opportunities as barriers to effective intervention delivery [28, 29]. Furthermore, they conveyed that GPs frequently misunderstood the scope of their roles and responsibilities, leading to an additional workload being shouldered by them [23]. This is echoed in several studies that also identified burnout issues among social prescribers.

#### Time management struggles

Social prescribers have indicated that balancing their social prescribing responsibilities with other tasks, such as devising strategic plans for individuals with special needs and complex health conditions, proves to be time-consuming [18, 21, 23, 28]. This complexity hampers their ability to stay visible to GPs and raise awareness of the service's existence among practice staff [23]. Furthermore, individuals grappling with issues such as anxiety, depression, and family bereavement demand specialised plans, further stretching the time allocation for social prescribers and presenting an additional challenge [22]. Additionally, the task of mapping local services and establishing connections with service providers, essential for addressing individuals' health and social needs, requires a significant investment of time and resources [21].

#### Impact of staff shortage and turnover

The continuity of individuals' participation in SP programs was disrupted by the high turnover of social prescribers [25, 27]. Those with long-term chronic conditions faced challenges in maintaining their health and well-being due to interruptions in support from exiting prescribers and establishing communication with new ones [26, 27, 29]. Apprehension about reaching out to new social prescribers hindered the smooth progression in the program [25]. Moreover, inconsistent intervention delivery approaches among social prescribers also impeded service utilisation [26]. One study [29] pointed out that short-staffing, stemming from staff allocations to various ongoing programs within the organisations, impacted SP delivery, and the scarcity of resources made recruiting new staff challenging.

#### Inadequate training and capacity building of social prescribers

Social prescribers have recognised their inadequate training to assess comprehensive needs and the referral process, which restricts their ability to address broader determinants of health [29, 30]. Inadequate supervision, both insufficient and ad hoc, was noted by coordinators, link workers, and service delivery personnel, which impeded effective service delivery [28, 29]. Moreover, the limited capacity of social prescribers to identify suitable services and the challenges in maintaining connections with community organisations have hindered the successful implementation of SP interventions [18, 29]. In certain studies, participants expressed that some social prescribers had limited or no healthcare background and lacked familiarity with culturally specific services to assist patients [18, 26]. Many studies highlighted the importance of developing the skills and capacity of social prescribers to effectively address patients' intricate physical and mental health issues, as well as to identify contextual changes in their lives that influence their well-being [22, 23, 26].

## External context and setting

### Opportunities

#### Availability of local community resources and partner organisations

In every study, researchers acknowledged the significance of accessibility to local community resources and the importance of partner organizations in delivering SP programs[8, 12, 18–30]. In a few studies, the practice of monthly PHC meetings involving social prescribers facilitated exchange of valuable information regarding existing resources and services, aiding social prescribers in compiling a comprehensive list of available resources/services [18, 21].

### Challenges

#### Accessibility and utilisation barriers

Barriers such as adverse economic conditions, travel-related time and costs [19], limited internet access [26], and minimal digital literacy [26] impeded the accessibility and utilisation of social prescriber services [8, 22]. Additionally, short intervention periods [12], unsafe intervention environments [19], unavailability of desired services [24, 26], strict schedules and inconvenient timing [19] were all identified as barriers. Engagement in SP programs was also challenged due to the unavailability of programs tailored to specific ages and genders, as young participants were directed to interventions designed for older individuals, and there was a scarcity of gender-specific exercise sessions [27]. Similarly, language barriers hindered service use for the black and minority ethnic groups. Culturally inappropriate services were also noted as a barrier, with participants facing challenges in adapting their diets and lifestyles to Westernised healthy eating practices [27].

#### Funding and resource constraints

A predominant issue revealed by most studies is the insufficiency and instability of funding, which emerges as a major barrier to the effective execution of SP programs. This scarcity of resources or funds further obstructed the recruitment and retention of skilled social prescribers. Moreover, GPs voiced concerns regarding the existence of unstructured SP programs constrained by limited time and funding. One study noted the impact of these challenges on the roles of both GPs and social prescribers in fulfilling their respective responsibilities [18].

## Discussion

To the best of our knowledge, this is the first review sought to understand and explore the existing SP programs for people living with long-term chronic conditions and to identify the opportunities and challenges in implementing such initiatives in PHC settings. The findings underscored a diverse array of recommended SP programs for individuals with various long-term chronic conditions, highlighting social prescribers' pivotal role in guiding the transition from PHC to other non-clinical services. Despite being a relatively nascent field, the literature highlights the burgeoning interest and significance of SP interventions for those with long-term chronic conditions.

This review identified a range of factors related to opportunities and challenges of implementation and delivery of SP programs for individuals living with long-term chronic conditions, which align with previous findings [32, 33]. Opportunities and challenges were related to workforce capacity to address holistic needs, availability and accessibility of community-based supportive initiatives or local services, implementation approach, staff turnover, relationships and communication, engagement and collaboration, funding and resources constraints. Social prescribers play a pivotal role in these initiatives, where their attributes serve as both facilitators and barriers, significantly impacting the success of SP programs [34, 35]. One identified impeding factor is the discrepancies in the literature regarding their roles and responsibilities, ranging from identifying social needs and setting goals to motivating patients and referring them to non-clinical services, both in paid and volunteer capacities. Emerging evidence underscores the necessity for a clear strategy that defines the skills, qualifications, pertinent training, accreditation, and role delineation for social prescribers before initiating and implementing SP programs[34, 36]. This review identified various facilitating skill sets of social prescribers, such as interpersonal skills, advocacy, cultural sensitivity, and support to encourage participation in social prescribing programs, closely mirroring what is reported in existing literature [37–39]. Conversely, insufficient capacity among social prescribers, often attributed to inadequate training and support, was identified as an important barrier in the current and previous studies [32, 35, 40–43]. Therefore, social prescribers require regular training and professional development plans to acquire or refresh these skill sets.

Prior studies have emphasised the advantages of supervision and mentoring in cultivating a supportive work environment [44, 45]. This approach mitigates job-related anxiety, improves skills and knowledge, and allows the application of feedback in tackling challenges in demanding work settings [44, 45]. Access to training, education, supervision, and consistent feedback is evidently linked to program sustainability, influencing staff-related outcomes such as turnover rates and job satisfaction [46–48]. Consequently, social prescribers require supervision and mentoring in the workplace to facilitate their professional growth and development.

Another identified barrier impacting the successful implementation of SP programs was uncertainties surrounding SP processes and procedures, coupled with high turnover rates among social prescribers. Therefore, when recruiting individuals for the role of a social prescriber, considering their experience and skills gained from previous occupations emerges as a valuable approach. The relevance of prior work experiences can include effective interpersonal communication, conducting needs assessments, managing client relationships, networking with service providers, and possessing a broad understanding of approaches to support individuals in addressing their holistic needs [27, 30, 33, 49]. Additionally, offering targeted training in mental health first aid and trauma response would enhance the capability of social prescribers to effectively assist people with both long-term chronic conditions and mental health concerns [50].

Various factors determined the SP referrals originating from PHC settings. The relationship between social prescribers, PHC staff teams, and other service providers significantly impacted the success of SP programs. Findings are consistent with previous qualitative studies indicating that the commitment of GPs and clinical leadership at the practice level can significantly influence the implementation of SP programs [51, 52]. In a study by Husk et al. [42], it was noted that the quality of healthcare providers' interactions with patients greatly impacts how referrals are received and how patients engage with the referred services. Evidence suggests that the visibility of social prescribers within general practices contributes to effective buy-in from health professionals, enabling them to make referrals to link workers they are familiar with and trust [35, 53]. To strengthen the social prescriber-GP relationship, SP programs could integrate social prescribers as an essential part of PHC staff teams and involve them in practice-level meetings [8].

Despite some studies used Well-being Star tool to assess the holistic needs and demonstrated well-defined referral procedures [12, 19, 22, 25–27, 30], many were unclear about the procedure to identify holistic needs and defined referral procedures. This finding is consistent with a study conducted by Moore et al. [35], which highlighted that paperwork and referral procedures can be time-consuming and confusing. Therefore, this review calls for the use or development of appropriate tools that capture the holistic needs of the people, which can be employed by social prescribers when assessing patients' needs. More importantly, the referral process needs to be clear enough for long-term chronic condition patients to access the referred services promptly. In line with our findings, we highlight the need for clear reporting of need assessment tools applied and documentation of the referral process in SP programs for people with long-term chronic conditions.

At the patient level, those managing multiple health conditions showed higher rates of absenteeism and disengagement from social prescribing interventions. The reduced involvement of such individuals in social prescribing may be attributed to the greater burden of managing multiple conditions, which encompass emotional and physical aspects [54]. Challenges in attending social referral appointments for various services [55] and the insufficient customisation of services by social prescribers for individuals' complex needs might also contribute to this disengagement. These findings highlight that service commissioners, funders, and researchers need to consider these factors when designing tailored social prescribing interventions for individuals with multiple health conditions.

People living with long-term chronic conditions often require a range of non-clinical support in addition to medical interventions that vary depending on factors such as the local context, the health literacy of patients, the models of care used to deliver services, and the accessibility and availability of local community assets or supportive initiatives. Emerging evidence highlights the value of adopting a “whole-system approach to SP that involves multiple organisations within a system offering community-based and non-clinical support, rather than relying solely on the role of an individual 'social prescriber' employed by a single organisation [56]. However, implementing and evaluating the 'whole-system model' in practice presents challenges. This is especially true without a clear understanding of the links, relationships, and referral pathways across organisations to discern what works well and what doesn't. Moving forward, there is a need to drive research in the field of social prescription. Establishing a community of practice to facilitate the exchange of best practices and ideas and high-quality evidence on systematic referral and follow-up processes will be instrumental in linking individuals to services and activities. This highlights the necessity for more rigorous research to evaluate different models of care for SP delivery to individuals with long-term chronic conditions. In the evaluation of these models, implementation researchers should place greater emphasis on a personalised, holistic approach to social prescribing rather than overly prescriptive social prescribing interventions, as the former is more likely to yield success.

Although the role of social prescribers in assisting individuals with multiple health conditions is gaining recognition globally, the formal evaluation of their effectiveness remains limited [21, 38, 39, 57]. Given the absence of a standardised evaluation framework for SP programs, this review underscores the requirement for a well-defined comprehensive implementation and evaluation framework. Such a framework should incorporate the perspectives of patients, healthcare practitioners, service providers and social prescribers to optimise intervention components. Developing such an evidence-based framework would minimise redundancy and contribute to a comprehensive knowledge base that can steer the development of effective SP intervention models for individuals with long-term chronic health conditions. While designing and implementing SP interventions for people with long-term chronic conditions from Indigenous and marginalised communities where health is not solely the physical well-being of an individual but refers to the social, emotional and cultural well-being of the whole community that helps individual to achieve full potential as a human being [58]; interventions should consider a holistic approach that addressed social, emotional, spiritual and cultural determinants of their health and well-being [59].

Another gap identified in the literature is the inadequate active stakeholder engagement in designing and implementing social prescribing interventions. Recent research underscores the significance of involving a broader range of stakeholders through "co-approaches in implementation science," encompassing co-design, co-production, and co-creation of initiatives. These iterative techniques enhance usability, prioritise people-centeredness, and facilitate real-world implementation while mitigating challenges [60]. Effectively addressing the intricate health and social needs of individuals with long-term chronic conditions necessitates coordinated care across primary care, social, community, and specialty services. This effective coordination can be achieved through transformational leadership, the cultivation of an inclusive environment, and the fostering of collaborative cultures within organisations [61]. These endeavours establish rapport with other service providers to holistically address needs [62], thereby cultivating trust and gaining stakeholder support for the proposed initiatives [63, 64]. Considering these elements, this review advocates for the SP program under a transformational leadership model [65], actively engaging diverse stakeholders in service design and execution.

### Strengths and limitations

Strengths of the present review include i) map of social prescription program initiatives for people with long-term conditions, (ii) the generation of evidence to guide future SP implementation work focusing on people with long-term chronic conditions and iii) the application of a scientific review approach with a slight modification of methodology to generate evidence.

We acknowledge that the findings in this review might not be comprehensive and could be subjected to publication bias, as we excluded published program reports, grey literature, and policy guidelines. Our search was limited to specific databases and terms, potentially overlooking overlooking articles indexed in other databases or using alternative search terms. Additionally, no quality appraisal was done for the included studies. Another limitation is that with the exception of one study, all other included studies were from the UK, and the perspectives of operational service managers, commissioners and funders are not represented in this research.

Also, as this was not a commissioned review, decision tools developed for commissioned reviews, such as SelecTing Approaches for Rapid Reviews (STARR) decision tool, were not applied. Furthermore, considering separate NHS systems in place for each of the four countries that make up the UK where the majority of the included studies originated with limited representation from other countries, caution is needed when applying these findings within the UK or elsewhere.

## Conclusion

In conclusion, effective implementation of the SP program hinges on several crucial factors, including adopting a holistic approach to need assessment, establishing clear referral pathways, ensuring the availability of local community resources and assets, fostering primary care leadership, and increasing awareness of the benefits of supportive services. Recognising the challenges and developing strategies to address them will pave the way for a more integrated and impactful approach to supporting individuals with long-term chronic conditions, ultimately improving the health and well-being outcomes of people with long-term chronic conditions through the PHC-centred SP program. To achieve this, it's essential to leverage the strengths of collaboration and coordination while addressing funding constraints and training needs. Healthcare systems must optimise the potential of SP interventions for long-term sustainability. Furthermore, researchers should value the local context when translating the insights from this study to a specific setting.

### Supplementary Information

**Additional file 1: Supplementary file 1.** Search terms and Boolean operators used across various databases.

**Additional file 2: Supplementary file 2.** Data extraction sheet .

## Data Availability

The relevant data used and/or analysed in this manuscript are provided as supplementary material.

## Abbreviations

CCGs: Clinical commissioning groups
COPD: Chronic obstructive pulmonary diseases
NHS: National health service
PHC: Primary health care
PRISMA: Preferred reporting items for systematic review and meta-analysis
SP: Social prescribing
WHO: World health organization
