# Supplementary material for: A rapid review of opportunities and challenges in the implementation of social prescription interventions for addressing the unmet needs of individuals living with long-term chronic conditions
Source: BMC Public Health. 2024 Jan 27;24:306. doi: 10.1186/s12889-024-17736-2 (PMC10821289; doi:10.1186/s12889-024-17736-2)
Supplement: Supplementary file 1 — Additional file 1: Supplementary file 1. Search terms and Boolean operators used across various databases. [file 12889_2024_17736_MOESM1_ESM.docx]

**Supplementary file.1. Search terms and Boolean operators used across various databases**

| **Web of Science** | ((((ALL=(social prescription OR social referral OR community referral OR community prescription OR social interventions OR social prescription OR linking scheme OR linking schemes OR social referrals )) AND ALL=(link worker OR social worker OR health care worker OR community health care worker OR navigator OR Aboriginal health worker OR Patient Navigator OR community health worker)) AND ALL=(primary health care OR health care OR primary care OR health practice OR general practice, health centres )) AND ALL=(Chronic disease OR multimorbidity OR cardiovascular disease OR diabetes OR Type 2 diabetes OR Chronic obstructive pulmonary disease OR respiratory difficulties OR heart disease OR asthma OR hypertension OR blood pressure OR osteoporosis OR chronic condition OR long-term conditions OR Obesity )) AND ((LA==“ENGLIS”) AND DT==“ARTICL”) AND DT==“ARTICL”) AND LA==“ENGLIS”) AND DT==“ARTICL”)) NOT (SE==“ANNALS OF THE NEW YORK ACADEMY OF SCIENCES SERIE”) OR CF==“ANNUAL MEETING OF THE AMERICAN GERIATRICS SOCIET” OR“19TH IAGG WORLD CONGRESS OF GERONTOLOGY AND GERIATRIC” OR“1ST ANNUAL INTERNATIONAL ASIAN CONFERENCE ON CANCER SCREENIN” OR“1ST INDIGENOUS CARDIOVASCULAR HEALTH CONFERENCE OF THE CARDIAC SOCIETY OF AUSTRALIA AND NEW ZEALAN” OR“1ST INTERNATIONAL CONFERENCE ON SOCIAL WORK IN HEALTH AND MENTAL HEALTH CARE” ))) |
| --- | --- |
| **Pubmed** | (Chronic disease OR multimorbidity OR cardiovascular disease OR diabetes OR Type 2 diabetes OR Chronic obstructive pulmonary disease OR respiratory difficulties OR heart disease OR asthma OR hypertension OR blood pressure OR osteoporosis OR chronic condition OR long-term conditions OR Obesity) AND ((primary health care OR health care OR primary care OR health practice OR general practice, health centres AND ((y_10[Filter]) AND (fft[Filter]) AND (english[Filter]))) AND ((social prescription OR social referral OR community referral OR community prescription OR social interventions OR social prescription OR linking scheme OR linking schemes OR social referrals) AND (link worker OR social worker OR health care worker OR community health care worker OR navigator OR Aboriginal health worker OR Patient Navigator OR community health worker) AND ((y_10[Filter]) AND (fft[Filter]) AND (english[Filter])))) |
| **Embase** | ( social prescription OR social referral OR community referral OR community prescription OR social interventions OR social prescription OR linking scheme OR linking schemes OR social referrals ) AND ( link worker OR social worker OR health care worker OR community health care worker OR navigator OR Aboriginal health worker OR Patient Navigator OR community health worker ) AND ( Chronic disease OR multimorbidity OR cardiovascular disease OR diabetes OR Type 2 diabetes OR Chronic obstructive pulmonary disease OR respiratory difficulties OR heart disease OR asthma OR hypertension OR blood pressure OR osteoporosis OR chronic condition OR long-term conditions OR Obesity ) AND ( primary health care OR health care OR primary care OR health practice OR general practice, health centres ) |
